# Clinical and Economic Impact of Community-Onset Urinary Tract Infections Caused by ESBL-Producing *Klebsiella pneumoniae* Requiring Hospitalization in Spain: An Observational Cohort Study

**DOI:** 10.3390/antibiotics10050585

**Published:** 2021-05-15

**Authors:** Dawid Rozenkiewicz, Erika Esteve-Palau, Mar Arenas-Miras, Santiago Grau, Xavier Duran, Luisa Sorlí, María Milagro Montero, Juan P. Horcajada

**Affiliations:** 1 Service of Infectious Diseases, Hospital del Mar, 08003 Barcelona, Spain; dawid.rozenkiewicz01@estudiant.upf.edu (D.R.); erika.esteve@pssjd.org (E.E.-P.); marenas@psmar.cat (M.A.-M.); lsorli@psmar.cat (L.S.); mmontero@psmar.cat (M.M.M.); 2 CEXS, Universitat Pompeu Fabra, Universitat Autònoma de Barcelona, 08003 Barcelona, Spain; 3 Infectious Pathology and Antimicrobials Research Group (IPAR), Institut Hospital del Mar d’Investigacions Mèdiques (IMIM), 08003 Barcelona, Spain; sgrau@psmar.cat; 4 REIPI, Instituto de Salud Carlos III (ISCIII), 28029 Madrid, Spain; 5 Pharmacy Service, Hospital del Mar, 08003 Barcelona, Spain; 6 Institut Hospital del Mar d’Investigacions Mèdiques (IMIM), 08003 Barcelona, Spain; xduran@imim.es

**Keywords:** ESBL-producing *Klebsiella pneumoniae*, urinary tract infection, clinical impact, economic impact

## Abstract

*Objective*: To analyze the clinical and economic impact of community-onset urinary tract infections (UTIs) caused by extended-spectrum beta-lactamase (ESBL)-producing *Klebsiella pneumoniae* requiring hospitalization. *Methods*: A retrospective cohort study that included all adults with a UTI caused by *K. pneumoniae* that were admitted to a tertiary care hospital in Barcelona, Spain, between 2011 and 2015. Demographic, clinical, and economic data were analyzed. *Results*: One hundred and seventy-three episodes of UTIs caused by *K. pneumoniae* were studied; 112 were non-ESBL-producing and 61 were ESBL-producing. Multivariate analysis identified ESBL production, acute confusional state associated with UTI, shock, and the time taken to obtain adequate treatment as risk factors for clinical failure during the first seven days. An economic analysis showed differences between ESBL-producing and non-ESBL-producing *K. pneumoniae* for the total cost of hospitalization per episode (mean EUR 6718 vs EUR 3688, respectively). Multivariate analysis of the higher costs of UTI episodes found statistically significant differences for ESBL production and the time taken to obtain adequate treatment. *Conclusion*: UTIs caused by ESBL-producing *K. pneumoniae* requiring hospitalization and the time taken to obtain adequate antimicrobial therapy are associated with worse clinical and economic outcomes.

## 1. Introduction

Extended-spectrum beta-lactamases (ESBLs) are enzymes produced by Gram-negative bacilli that inactivate oxyimino beta-lactam antibiotics (cephalosporins and aztreonam), but not cephamycins (cefoxitin) or carbapenems. They are generally plasmid-mediated and are derived from other enzymes with a narrower spectrum of hydrolysis. Al-though many species of Gram-negative bacilli can produce ESBLs, *Escherichia coli* and *Klebsiella pneumoniae* are the major ESBL producers. According to the WHO, ESBL-producing *Enterobacterales* must be regarded as critical priority pathogens due to their resistance to antibiotics [1]. ESBL-producing bacteria are a major cause of both community-based and healthcare-associated infections and are globally disseminated, although their incidence varies in different parts of the world [2]. An increase in community-acquired ESBL-producing *E. coli* and *K. pneumoniae* has been recently reported [3]. *K. pneumoniae* is associated with pneumonia, urinary tract infections (UTIs), intra-abdominal infections and sepsis [4]. Data on the incidence of UTIs in Spain place *K. pneumoniae* as the second cause of UTIs of community and nosocomial origin [5].

ESBL-producing *K. pneumoniae* has been considered almost exclusively as a nosoco-mial pathogen due to its epidemiological behavior, although recent data show that it is also an important agent involved in processes of community origin [6]. Data from a multicenter study conducted in 11 hospitals in Spain from 2011 to 2016 showed an overall increase in ESBL-producing *K. pneumoniae,* compared to a similar study covering the 2002–2010 period, reaching a frequency of more than 18% in 2016. In that study, ESBLs were more prevalent in *K. pneumoniae* (16.3%) and *E. coli* (9.5%) isolates of nosocomial origin, followed by community-acquired *K. pneumoniae* (9.5%) [7].

Infections produced by ESBL-producing microorganisms pose important therapeutic challenges. The fact that ESBL-producing bacteria are resistant to all penicillins and cephalosporins, including third- and fourth-generation ones, means that infections due to these bacteria have limited therapeutic options [8]. As a result, infections caused by ESBL-producing bacteria can lead to increased mortality, an increased length of hospital stay, and higher hospital costs compared with infections caused by non-ESBL-producing bacteria of the same species [9,10]. The same phenomenon tends to be significantly stronger among patients with ESBL-producing *K. pneumoniae* infections compared with those with ESBL-producing *E. coli* infections [11].

A study was recently carried out to assess the clinical impact and consumption of health resources among patients with community-onset UTIs due to ESBL-producing *E. coli* admitted to our hospital. In that study, the presence of ESBL among *E. coli* strains was associated with higher clinical failure rates in the first seven days, as well as higher economic costs [12].

Bearing in mind that UTIs place an economic burden on both society and the healthcare system, that *K. pneumoniae* is a frequent cause of UTIs, and that ESBL-producing *Enterobacterales* cannot be considered a homogeneous group, the objective of this study was to analyze the clinical and economic impact of ESBL-producing *K. pneumoniae* as a cause of UTI in patients requiring admission to our hospital.

## 2. Results

One hundred and seventy-three UTI episodes met the criteria for inclusion during the study period and were included; 112 were due to non-ESBL-producing *K. pneumoniae* and 61 were due to ESBL-producing *K. pneumoniae*. The baseline characteristics of patients, broken down into those with and without ESBL infections, are shown in Table 1.

The bivariate analysis showed a significantly higher prevalence of men, AHA-UTIs, and previous antibiotic use (especially quinolones and cephalosporins) in the ESBL group. The clinical characteristics and procedures carried out on the studied patients, comparing ESBL and non-ESBL infections, are shown in Table 2. Among the admissions caused by ESBL-producing *K. pneumoniae*, there was a significantly higher clinical prevalence of cystitis; more frequent clinical failure at seven days; a longer time taken to obtain adequate treatment; longer hospitalization; and a more frequent use of infectious disease specialist consultants, pharmacy intervention, and home hospitalization. Among non-ESBL-producing *K. pneumoniae* infections, there was a more frequent use of adequate empirical antibiotics and a higher number of positive blood cultures, cases of pyelonephritis, and cases of sepsis.

In the non-ESBL-producing group, the most commonly used empiric antibiotics were cephalosporins (32.1%), amoxicillin/clavulanate (30.4%), and carbapenems (11.6%); adequate coverage was achieved in 92.9% of cases. In the ESBL-producing group, the most commonly used empiric treatments were amoxicillin/clavulanate (26.2%), cephalosporins (19.7%), and carbapenems (18%), and adequate coverage was achieved in only 37.7% of cases. The antibiotics most commonly used as directed therapy in the non-ESBL-producing group were ciprofloxacin (45.5%) and amoxicillin/clavulanate (19.6%), and, in the ESBL-producing group, ertapenem (45.6%) and imipenem (29.5%).

Table 3 shows the multivariate analysis of factors associated with clinical failure at seven days. ESBL production, the acute confusional state associated with a UTI, shock, and the time taken to obtain adequate therapy were factors independently associated with clinical failure at seven days.

An analysis of economic data can be found in Table 4. The analysis showed a mean difference of EUR 3.030 between the two groups for the total cost of hospitalization in favor of the ESBL-producing group. The costs associated with medication, nursing, and antibiotics accounted for this difference.

Table 5 shows the multivariate analysis of costs. The presence of ESBL, shock at admission, time taken to obtain adequate treatment, and the length of hospitalization were variables independently associated with higher hospitalization costs.

We performed a post-hoc power calculation to compare the median of the cost of hospitalization between non-ESBL and ESBL groups. The power of the study for comparing the costs was 1.0.

## 3. Discussion

The present study showed that the clinical outcomes were worse and the hospital costs were higher for community-onset UTIs caused by ESBL-producing *K. pneumoniae* requiring hospitalization compared to the UTIs caused by non-ESBL-producing *K. pneumoniae*. These findings confirm that ESBL *Enterobacterales* are, as the WHO states, critical priority pathogens, adding more support for this argument. [1].

There are several risk factors for the acquisition of a UTI caused by ESBL-producing microorganisms, including healthcare contact, previous antibiotic use, recurrent UTIs, a urinary catheter, old age, and male gender [13]. Our study identified a higher prevalence of ambulatory healthcare-associated infections, previous antibiotic use, and male gender in the ESBL-producing group. Ambulatory healthcare-associated UTIs have previously been identified as more frequently caused by antibiotic-resistant microorganisms than community-acquired UTIs and have important clinical consequences [13]. Higher male gender prevalence could be attributed to the fact that UTIs in males tend to have more hospitalization criteria. These findings are similar to those from a recent study from Denmark [14].

There was a significant difference in the clinical response (22.4%) between the two groups, with lower response rates in the ESBL-producing group, similar to previous studies performed with *E. coli* [11]. A recent study was also carried out to assess the clinical impact and consumption of health resources among patients with community-onset UTIs due to ESBL-producing *E. coli* admitted to hospital. In that study, the presence of ESBL among *E. coli* strains was associated with higher clinical failure rates in the first seven days, as well as higher economic costs. Similar to the present study, in that study ESBL production was significantly related to clinical failure, and mean differences in the cost of hospitalization were EUR 2,368 [12]. Other variables associated with a worse clinical response were clinical presentation with a confusional state and shock. The time taken to obtain adequate antimicrobial therapy was also an independent factor associated with a worse clinical response. Previous studies have shown that patients who received adequate empirical treatment were more likely to have a better clinical course [15]. Hospital stay was significantly longer in ESBL-UTI patients. This is another important clinical consequence that could probably be avoided or reduced by improving initial or early antimicrobial management. Our data confirm that inadequate antimicrobial therapy is associated with a worse prognosis for patients with *K. pneumoniae* UTIs requiring hospital admission without differences in mortality. These findings are similar to those from a recent study from Denmark [16]. Infectious diseases specialist consultations or pharmacy interventions were more frequent in ESBL episodes. However, in the multivariate analysis of the clinical response at day 7, these interventions were not statistically significant, probably because they have an influence on the directed antimicrobial therapy, and not on the empirical therapy.

Interestingly, we observed that among patients with ESBL-producing *K. pneumoniae* infections, there was a higher prevalence of cystitis, whereas among patients with non-ESBL-producing *K. pneumoniae* UTIs clinical symptoms of pyelonephritis, positive blood cultures, sepsis, and ICU admission were more prevalent. These data seem to indicate a lower virulence in ESBL strains, a phenomenon that has already been reported in other studies involving *E. coli*, in which the acquisition of quinolone resistance was associated with a loss of virulence factors [17]. The relationship between resistance and bacterial virulence has also been studied in recent years. For instance, the acquisition by *E.coli* of the OXA-10, OXA-24 or SFO-1 family of beta-lactamases is known to be associated with a loss of virulence due to alterations in the formation of peptidoglycan, probably caused by residual enzymatic activity in the β-lactamases, similar to that of penicillin-binding proteins [18]. In the case of *K. pneumoniae*, there are studies describing the role of mechanisms such as the deletion of ompK36 and ompK36 porins in the loss of virulence and the acquisition of resistance [19]. Our cohort was comprised of community-acquired and ambulatory healthcare-associated UTIs, which are environments in which the selective pressure of antibiotics is not as high as in the nosocomial setting. It is likely that the loss of virulence in these environments due to the acquisition of resistance is better tolerated by microorganisms.

With respect to the economic impact, we showed that the total hospitalization costs associated with patients with ESBL-producing *K. pneumoniae* UTIs admitted to hospital were almost double those of patients with non-ESBL-producing *K. pneumoniae* UTIs. The cost of medication and the nursing costs accounted for the difference in total cost. The difference in medication cost was mainly due to the use of more expensive antibiotics such as carbapenems. The time taken to obtain adequate treatment was also identified as a variable related to the increased total cost of hospitalization and was probably an indirect effect of a worse clinical response, as seen in previous studies [9,12].

The main limitation of our study was its retrospective design. However, this design permitted the study of a higher number of patients in less time.

## 4. Materials and Methods

A retrospective cohort study was conducted from January 2011 to January 2016 at the Hospital del Mar, a tertiary university hospital with 420 beds serving a population of 340,000 people in the city of Barcelona (Spain). The study included all adults (older than 17 years) admitted to the Hospital del Mar during the study period with urinary tract infections and a urine culture that tested positive for *K. pneumoniae*. The only UTI origins considered were strictly community-acquired (CA) and ambulatory healthcare-associated (AHA). Hospital-acquired UTIs were excluded. In cases of multiple episodes requiring admission, only the first episode was studied. UTIs caused by microorganisms other than *K. pneumoniae*, cultures showing mixed flora, and patients with asymptomatic bacteriuria were excluded.

### 4.1. Variables

Patient data were collected retrospectively from hospital electronic medical records. The following variables were collected: demographic and epidemiological factors (age, gender, underlying diseases, use of immunosuppressive therapy, prior antibiotic treatment), clinical and microbiological data (UTI symptoms, sepsis, shock, empirical and definitive antibiotic treatment, time to obtain adequate treatment, clinical response at seven days, infectious diseases and/or pharmacy services interventions, ICU admission, emergency room visits after discharge, hospital readmissions, convalescent or subacute hospitalization after discharge, mortality at 30 days), and risk factors for ESBL-producing *K. pneumoniae* (urinary catheter, urological manipulation, urologic conditions, type of acquisition: community-acquired (CA) versus ambulatory healthcare-associated (AHA). The Charlson Index was used to classify comorbidities [20] and the McCabe–Jackson index was used to classify their severity [21]. The main variable used to analyze the clinical impact was clinical response seven days after admission. The variables selected to study the use of clinical resources were: duration of hospitalization, cost of hospitalization, use and cost of antibiotic treatment, emergency room visits, use of home hospitalization and the need for re-admission after 30 days. The costs of hospitalization were obtained from the hospital database and were broken down into cost of medication, cost of antibiotics, cost of nursing, laboratory costs, radiology costs, pharmacy costs, specialist consultations and emergency visits.

### 4.2. Definitions

A diagnosis of symptomatic UTI was established if the patient presented with one of the following signs or symptoms: a fever of >38 °C, urinary urgency, polyuria, dysuria or suprapubic pain, and a positive urine culture (more than 10^5^ CFU of uropathogen per mL of urine). Five UTI syndromes were considered:

1. Cystitis: the presence of dysuria, urinary frequency, urgency and occasionally hematuria in patients without fever (axillary temperature < 38 °C).
2. Pyelonephritis: the presence of fever (axillary temperature > 38 °C) and spontaneous lumbar pain or pain on costovertebral percussion, with or without increased urinary frequency, dysuria or urine retention.
3. Acute prostatitis: a sudden febrile episode in men accompanied by lower back and perineal pain with polyuria or dysuria, and/or urinary retention.
4. The confusional state associated with UTIs was defined as an episode of confusion attributed to an underlying UTI after excluding other infectious foci and other causes.
5. Urinary sepsis: a systemic inflammatory response syndrome with a positive urine culture or blood culture for an uropathogen with no other apparent source of infection [22].

A CA-UTI was defined as a UTI detected within the first 48 h of hospital admission that did not meet the criteria for an AHA-UTI. An AHA-UTI was defined as a UTI detected within the first 48 h of hospital admission and met one of the following criteria [14]:

1. The patient had received specialized treatment at home by qualified healthcare workers within 30 days prior to hospital admission.
2. The patient had attended a day hospital, hemodialysis clinic or had received intravenous chemotherapy within 30 days prior to hospital admission.
3. Hospitalization for more than 48 h during the 90 days preceding the current admission.
4. Resident in a long-term care facility or nursing home.
5. The patient had undergone an invasive urinary procedure within 30 days of the episode or had a long-term indwelling urethral catheter.

A community-onset UTI was defined as any CA-UTI or AHA-UTI.

Previous antibiotic therapy was defined as the use of antibiotics in the three months prior to the diagnosis of a UTI. Empirical therapy was administered before the in vitro susceptibility of the uropathogen that caused the episode was known. Empirical therapy was considered inadequate if the microorganism causing the UTI was not fully susceptible to the antibiotic used.

The response to treatment at seven days was considered satisfactory if the patient was asymptomatic or there was a significant improvement in the signs and symptoms of infection; the treatment response was unsatisfactory if there was persistence or progression of signs and symptoms of infection, if a change in pharmacological agent was required after three days of treatment, or if an infection-related death occurred.

### 4.3. Statistical Analysis

Data from patients with and without ESBL-producing *K. pneumoniae* were compared. Quantitative variables were compared using the Student’s *t*-test or the Mann–Whitney U test if the distribution of the data was not normal. Qualitative variables were compared using the Chi-square test. Bivariate and multivariate analyses were performed to elucidate the variables independently related to clinical failure at seven days and to higher hospital costs. The clinical response was analyzed by binary logistic regression. Multivariate median regression models were conducted through the forward stepwise approach. This was performed in terms of statistical signification of coefficients of variables (*p* < 0.1), but also to ensure clinical consistency of variables included in the model.

With respect to hospital costs, the normality of the variables was assessed by histogram inspection, testing for normality with a QQ-plot, and applying the Shapiro–Wilk W–test. Once non-normality was established (*p* < 0.001), hospital costs were analyzed by median regression. Associations with *p*-values < 0.05 were regarded as statistically significant. Statistical analyses were performed using the SPSS v.22 and STATA v.15.1 packages.

## 5. Conclusions

In conclusion, our study shows that the production of ESBL by *K. pneumoniae* causing community-onset UTIs and the time taken to obtain adequate antimicrobial therapy are independent factors for a worse clinical response and higher healthcare costs. It is important to identify risk factors in order to categorize these patients earlier so that they benefit from the appropriate empirical treatment that leads to a better clinical response and reduced hospitalization costs. Rapid diagnostic tests are also important in this scenario.

More studies of infections caused by multidrug-resistant bacteria in the community should be performed in order to prevent acquisition and optimize the management of infections in an attempt to reduce their clinical and economic burden.

## Figures and Tables

**Table 1:** Univariate analysis of patient characteristics.

	Non-ESBL	ESBL	*p*-Value *
Total	112	61	
Sex, male	29 (25.9%)	28 (45.9%)	0.011
Age (in years)	72.8 ± 18.8	75.8 ± 12.1	0.200
AHA-UTI	34 (30.1%)	38 (63.3%)	<0.001
Diabetes mellitus	50 (44.6%)	30 (49.2%)	0.633
Dementia	28 (25%)	13 (21.3%)	0.709
Immunosuppressive treatment	40 (35.7%)	25 (41%)	0.515
McCabe Index	2.38 ± 0.67	2.4 ± 0.64	0.105
Charlson Comorbidity Index Urinary catheterization	6.02 ± 2.7 8 (7%)	6.54 ± 2.12 10 (16.4%)	0.198 0.070
Other urinary catheters	9 (8%)	3 (4.9%)	0.543
Previous urological manipulation	16 (14.3%)	11 (18%)	0.519
Urological pathology	36 (32.1%)	22 (36.1%)	0.617
Kidney transplant	7 (6.3%)	2 (3.3%)	0.496
History of recurrent UTIs	39 (34.8%)	25 (41%)	0.510
History of pyelonephritis	16 (14.3%)	7 (11.5%)	0.648
Urinary incontinence	14 (12.5%)	14 (23%)	0.860
Previous antibiotic	38(33.9%)	39(63.9%)	<0.001
Amoxicillin/clavulanic acid	20 (17.9%)	10 (16.4%)	0.808
Trimethoprim/sulfamethoxazole	1 (0.9%)	2 (3.3%)	0.284
Quinolones	7 (6.3%)	10 (16.4%)	0.032
Fosfomycin	4 (3.6%)	3 (4.9%)	0.698
Cephalosporin	2 (1.8%)	6 (9.8%)	0.024
Carbapenems	1 (0.9%)	4 (6.6%)	0.053
Aminoglycosides	0 (0%)	1 (1.6%)	0.353
Linezolid	2 (1.8%)	1 (1.6%)	1.000
Others	1 (0.9%)	2 (3.3%)	0.284

* Student’s *t*-test, or Mann–Whitney U was used for comparing quantitative variables. The Chi-square test was used for comparing qualitative variables. ESBL: extended spectrum betalactamases; AHA-UTI: ambulatory Health Care-Associated Urinary Tract Infection. HCA-UTI: Health Care-Associated Urinary Tract Infection. CA-UTI: Community-Acquired Urinary Tract Infection.

**Table 2:** Univariate analysis of clinical data.

	Non-ESBL	ESBL	*p*-Value *
Cystitis	17 (15.2%)	23 (37.7%)	0.001
Pyelonephritis	39 (34.8%)	12 (19.7%)	0.038
Confusion syndrome associated with UTIs	34 (30.4%)	21 (34.4%)	0.611
Prostatitis	6 (5.4%)	2 (3.3%)	0.714
Sepsis	40 (35.7%)	12 (19.7%)	0.037
Shock	6 (5.4%)	0 (0%)	0.091
Positive blood culture	45 (40.2%)	13 (21.3%)	0.012
Infectious diseases intervention	32 (28.6%)	43 (70.5%)	<0.001
Pharmacy intervention	9 (8%)	13 (21.3%)	0.017
Appropriate empirical treatment	104 (92.9%)	23 (37.7%)	<0.001
Time to adequate treatment (days)	0.54 ± 1.4	1.59 ± 2.1	<0.001
Duration of hospital treatment (days)	4.57 ± 2.64	4.06 ± 4.1	0.956
Clinical response at seven days	82 (73.2%)	31 (50.8%)	0.004
Days of hospitalization	8.43 ± 6.42	11.62 ± 7.1	0.003
Readmission for the same UTI	24 (21.4%)	17 (27.9%)	0.355
Emergency consultation ^a^	25 (22.3%)	18 (29.5%)	0.358
Home hospitalization	5 (4.5%)	10 (16.4%)	0.011
ICU admission	6 (5.4%)	0 (0%)	0.091
Mortality within 30 days	12 (10.7%)	3 (4.9%)	0.263

^a^ Re-consultation in the emergency room within 30 days after discharge; * Student’s *t*-test or Mann–Whitney U was used for comparing quantitative variables. The Chi-square test was used for comparing qualitative variables. ESBL: extended spectrum betalactamases; ICU: intensive care unit.

**Table 3:** Univariate and multivariate analysis of factors associated with clinical failure at seven days.

	OR (95%CI)	*p*-Value *	Adjusted OR	*p*-Value **
Sex, male	1.230 (0.626,2.415)	0.548	1.182 (0.501,2.785)	0.702
Age > 77	1.155 (0.886,1.505)	0.286	1.129 (0.856,1.487)	0.388
Infectious diseases intervention	1.676 (0.891,3.154)	0.109		
Pharmacy intervention	1.683 (0.681,4.161)	0.259		
Previous antibiotic	1.314 (0.700,2.465)	0.396	1.763 (0.756,4.114)	0.189
Immunosuppressive treatment	2.001 (1.052,3.805)	0.034		
Urinary catheterization	0.505 (0.159,1.609)	0.248		
Other catheters	0.157 (0.020,1.248)	0.080		
Previous urological manipulation	0.280 (0.092,0.851)	0.025		
Urological pathology	0.543 (0.270,1.089)	0.086		
History of recurrent UTIs	1.092 (0.572,2.084)	0.790		
Diabetes	0.679 (0.360,1.280)	0.231		
Urological neoplasms	0.176 (0.051,0.609)	0.006		
McCabe–Jackson Index > 2	0.899 (0.684,1.181)	0.446	1.108 (0.848,1.447)	0.451
Charlson Comobilidity Index > 5.8	1.062 (0.862,1.308)	0.571	1.695 (0.921,3.120)	0.090
Bacteremia	1.936 (1.006,3.724)	0.048	2.412 (0.351,16.538)	0.370
Cystitis	1.018 (0.485,2.138)	0.962	1.656 (0.562,4.877)	0.360
Pyelonephritis	0.410 (0.192,0.875)	0.021	0.782 (0.246,2.487)	0.678
Prostatitis	0 (0,0)	0.999		
Confusion syndrome associated with UTIs	2.215 (1.142,4.296)	0.019	5.155 (1.670,15.906)	0.004
Sepsis	2.275 (1.163,4.451)	0.016	3.758 (0.519,27.208)	0.190
Shock	3.964 (0.705,22.306)	0.118	7.239 (1.008,51.983)	0.049
ESBL	2.645 (1.376,5.084)	0.004	2.622 (1.086,6.328)	0.032
Time to adequate treatment	1.359 (1.092,1.692)	0.006	1.364 (1.059,1.755)	0.016
Appropriate empirical treatment	0.217 (0.106,0.443)	<0.001		
Duration of hospital treatment	1.285 (1.161,1.422)	<0.001		
CA-UTI	0.997 (0.528,1.881)	0.993		

* Univariate analysis: Student’s *t*-test or Mann–Whitney U used for comparing quantitative variables. The Chi-square test was used for comparing qualitative variables. ** Multivariate analysis: binary logistic regression through the forward stepwise approach; ESBL: extended spectrum betalactamases; CA-UTI: Community-Acquired Urinary Tract Infection; OR: odds ratio.

**Table 4:** Univariate analysis of the economic impact of ESBL and non-ESBL-producing *K. pneumoniae* in Euros.

	Non-ESBL med[P_25_,P_75_]	ESBL med[P_25_,P_75_]	*p*-Value *
Cost of hospitalization	3688 [1783,4141]	6718 [3322,9611]	<0.001
Cost of pharmacy	457 [174,577]	888 [325,1158]	0.001
Cost of antibiotics	47 [7,31]	380 [87,544]	<0.001
Cost of nursery	1809 [880,2294]	4581 [2375,6630]	<0.001
Cost of laboratory	165 [39,201]	171 [81,192]	0.852
Cost of radiology	94 [0,111]	62 [1,72]	0.205
Cost of inter consultations	60 [0,33]	65 [0,71]	0.818
Cost of Emergency Room visits	341 [0,760]	463 [0,663]	0.218

* Student’s *t*-test or Mann–Whitney U. ESBL: extended spectrum betalactamases; med: median; P_25_:1st quartile; P_75_:3rd quartile

**Table 5:** Univariate and multivariate analysis of the cost of the UTI episode.

	DM (95%CI)	*p*−Value *	Adjusted DM	*p*−Value **
ESBL	3446 (2330,4561)	<0.001	2569 (993,4144)	0.002
Sex, male	417 (−1126,1960)	0.594	−285 (−1578,1008)	0.663
Age > 77	−117 (−727,373)	0.526	−101 (−1578,1008)	0.602
Infectious diseases intervention	2718 (1582,3854)	<0.001		
Pharmacy intervention	2977 (814,5140)	0.007		
Previous antibiotic	957 (−592,2508)	0.224		
Immunosuppressive treatment	2304 (703,3904)	0.005		
Previous urological manipulation	−1202 (−3073,668)	0.206		
Urological pathology	−1353 (−2706,−1)	0.050		
History of recurrent UTIs	647 (−1013,2308)	0.442		
Urological neoplasms	−1648 (−3347,51)	0.057	−413 (−1953,1127)	0.596
McCabe–Jackson Index > 2	39 (−421,501)	0.865		
Charlson Comorbidity Index > 5.8	0.56 (−714,715)	0.999		
Bacteriemia	2031 (114,3949)	0.038	1617 (−762,3996)	0.181
Cystitis	1847 (115,3579)	0.037		
Pyelonephritis	401 (−1371,2173)	0.655		
Prostatitis	−1653 (−4984,1677)	0.328		
Sepsis	1772 (−144,3690)	0.070	−479 (−2944−1985)	0.701
Shock	8750 (4680,12820)	<0.001	6812 (3925−9699)	<0.001
Others	−413 (−2001,1174)	0.608		
Time to adequate treatment	612 (117,1107)	0.016	546 (82,1010)	0.021
Appropriate empirical treatment	−3552 (−4928,−2177)	<0.001	157 (−1635,1950)	0.862
Duration of hospital treatment	494 (298,629)	<0.001	266 (140,392)	<0.001
CA−UTI	−827 (−2371,717)	0.291	788 (−1101,1391)	0.818

* Univariate analysis: Student’s *t*-test or Mann–Whitney U were used for comparing quantitative variables. The Chi-square test was used for comparing qualitative variables. ** Multivariate analysis: binary logistic regression through the forward stepwise approach; ESBLE: extended spectrum betalactamases; DM: difference between the median of the group that presents the variable and the median of the group that does not present it. The positivity of the value indicates an increase in the cost in the presence of the variable and the negativity decrease in the cost. CA-UTI: Community-Acquired Urinary Tract Infection.

## Data Availability

Not applicable.
